# Interpretable prediction of gross motor coordination in children aged 9–10 using machine learning and SHAP: the influence of physical fitness, basic coordination, and executive function

**DOI:** 10.7717/peerj.20827

**Published:** 2026-02-26

**Authors:** Lingfeng Mao, Yuan Sui, Xiangyang Ding, Min He, Liqin Deng, Yue Shi, Fei Li

**Affiliations:** 1 Shanghai University of Sport, School of Athletic Performance, Shanghai, China; 2 Shanghai University of Sport, Key Laboratory of Exercise and Health Sciences of Ministry of Education, Shanghai, China; 3 Shanghai University of Sport, Key Lab of Human Performance, Shanghai, China

**Keywords:** Gross motor coordination, Basic coordination capacity, Physical fitness, Machine learning, SHAP

## Abstract

**Background:**

Gross motor coordination is a fundamental component of children’s physical development and motor skill acquisition, closely associated with physical fitness, cognitive function, and overall health. This study aimed to examine the influence of physical fitness, basic coordination, and executive function (EF) on gross motor coordination, and to evaluate the predictive performance of machine learning models compared with traditional multiple linear regression (MLR).

**Methods:**

A total of 167 children (85 boys and 82 girls), aged 9–10 years, participated in the study. Gross motor coordination was assessed using the Körperkoordinationtest für Kinder (KTK). Physical fitness (*e.g*., 50 m sprint, standing long jump, sit-ups), basic coordination (*e.g*., kinesthetic differentiation, spatial orientation, balance), and EF (*e.g*., inhibitory control, working memory) were measured as predictors. Model performance was evaluated using R^2^, root mean square error (RMSE), and mean absolute error (MAE). SHapley Additive exPlanations (SHAP) were applied to interpret the best-performing model and analyze feature importance and nonlinear effects.

**Results:**

Among the models, Random Forest Regression (RFR) achieved the highest performance (*R*^2^ = 0.533, RMSE = 6.075, MAE = 4.850). SHAP analysis revealed that spatial orientation, body mass index (BMI), dynamic balance, standing long jump, and closed-eye balance were the most important predictors, with spatial orientation, BMI, and closed-eye balance showing notable nonlinear effects. EF contributed minimally to prediction.

**Conclusion:**

Spatial-body integration, physical fitness, and postural control are primary determinants of gross motor coordination in children, while cognitive regulation plays a secondary role. Training programs aiming to enhance gross motor coordination should emphasize spatial orientation, body weight management, balance, and lower-limb strength.

## Introduction

As a fundamental component of motor competence, gross motor coordination (GMC) supports essential large-movement tasks such as running, jumping, and balancing by reflecting the efficient interaction among the musculoskeletal, nervous, and sensory systems, allowing the body to perform precise and balanced movements with minimal energy expenditure (Gallahue & Ozmun, 2006; Latash & Lestienne, 2006). Previous studies have demonstrated that GMC is strongly associated with not only physical activity participation, self-care ability, and physical health, but also emotional regulation, social adaptation, and academic performance (Hulteen et al., 2018; Lopes et al., 2011; Rivilis et al., 2011). Children with lower GMC typically experience greater difficulties with motor tasks, daily activities, and school or social interactions (Cummins, Piek & Dyck, 2005; Fernandes et al., 2016). Moreover, approximately 9–28% of children and adolescents aged 10–12 present with GMC difficulties or related disorders (Tsiotra et al., 2006). During childhood, GMC directly or indirectly affects physical health and shapes long-term health trajectories (Cattuzzo et al., 2016). Accordingly, most experts advocate early interventions to enhance GMC in children. The age of 6–12 are considered a critical window for GMC development (Wade & Whiting, 1986), as delays during this period may hinder the acquisition of motor skills and negatively impact long-term physical and psychological health (Lopes et al., 2011).

The development of GMC in children is shaped by a constellation of factors, including physical fitness components (*e.g*., speed, strength, and flexibility) (Dos Santos et al., 2018), basic coordination capacities (BCC) such as kinesthetic differentiation, spatial orientation, balance, rhythm, and motor reaction as well as executive function (Silvestri et al., 2025). Although many studies have demonstrated the significance of these factors, their relative contributions to GMC remain inconclusive (D’Hondt et al., 2014; De Chaves et al., 2016; Fernandes et al., 2016; Freitas et al., 2015; Giuriato et al., 2021; Sui et al., 2024). Importantly, GMC development is not a linear process but rather has been shown to emerge from dynamic and multifactorial interactions, as studies have found that body mass index (BMI) shows a U-shaped relationship with GMC, and that the effects of spatial orientation and balance exhibit nonlinear patterns depending on ability levels (Dos Santos et al., 2018; Sui et al., 2024). However, most existing studies have relied on traditional statistical methods—correlation analysis, one-way ANOVA, and multiple linear regression (D’Hondt et al., 2013; De Chaves et al., 2016; Giuriato et al., 2021), which generally assume linear and additive relations and are therefore limited in capturing the complex, nonlinear mechanisms underlying GMC. Consequently, linear models may understate or mischaracterize these associations (Schober & Vetter, 2021). To better elucidate the predictive mechanisms of GMC, more flexible, expressive modeling.

In recent years, machine learning (ML) methods have shown demonstrated significant advantages over traditional statistics approaches in handling nonlinear, multivariable, and high-dimensional data. ML has been widely applied in domains such as health behavior prediction, early risk identification, and personalized intervention (Ali et al., 2021; Mandorino, Clubb & Lacome, 2024). At the same time, the game theory-based interpretability framework SHapley Additive exPlanations (SHAP) quantifies the contribution of each feature to the model’s predictions, providing both global and local interpretability (Lundberg et al., 2018; Lundberg & Lee, 2017). This makes complex models easier to understand and enhances the transparency and explanatory power of the model. SHAP analysis has already been widely applied in analyzing disease risk factors and running performance (Knechtle et al., 2025; Wang et al., 2021). Nevertheless, in the current research landscape of children’s GMC, the systematic application of machine learning and interpretable modeling techniques remains limited—particularly regarding model comparison and the identification of influential predictors.

To address this gap, the present study drew on data from 167 children aged 9–10 years, including measures of physical fitness, basic coordination capacities, and executive function. We modeled GMC performance using ML algorithms alongside multiple linear regression (MLR) and compared their predictive performance. While also applying SHAP analysis to interpret feature importance and probe potential nonlinear associations. By combining predictive accuracy with interpretability, this study improves GMC modeling in children and supports early identification and individualized interventions for coordination deficits.

## Materials and Methods

### Participants

Children aged 9–10 years were recruited using a convenience sampling method from three public primary schools in Shanghai, China (see Table 1 for descriptive statistics). Inclusion criteria were: (1) age between 9 and 10 years; (2) physical and cognitive ability to complete all assessments; (3) normal intellectual development; and (4) absence of congenital limb deformities or psychiatric disorders. Exclusion criteria were: (1) missing or abnormal data in any test; (2) limb injuries within 30 days prior to assessment; and (3) diagnosed conditions such as intellectual disability, muscular dystrophy, or cardiovascular disease. In total, 167 children (85 boys, 82 girls) met eligibility and were included, yielding a balanced sex distribution. The study was approved by the Shanghai University of Sport Ethics Committee (Approval No. 102772023RT108). Written informed consent was obtained from guardians, and verbal assent from children.

**Table 1:** Descriptive statistics of basic information.

	Variable	Male (*n* = 85)	Female (*n* = 82)	Total (*n* = 167)
Basic information	Age	9.47 ± 0.5	9.51 ± 0.5	9.49 ± 0.50
	Height (m)	1.39 ± 0.07	1.41 ± 0.07	1.40 ± 0.07
	Weight (kg)	38.72 ± 9.5	34.98 ± 8.1	36.89 ± 9.02
	Leg length (cm)	71.74 ± 6.57	73.90 ± 5.75	72.80 ± 6.26
	BMI	19.73 ± 3.82	17.53 ± 2.80	18.65 ± 3.52

### Assessment procedures

**Figure 1:**
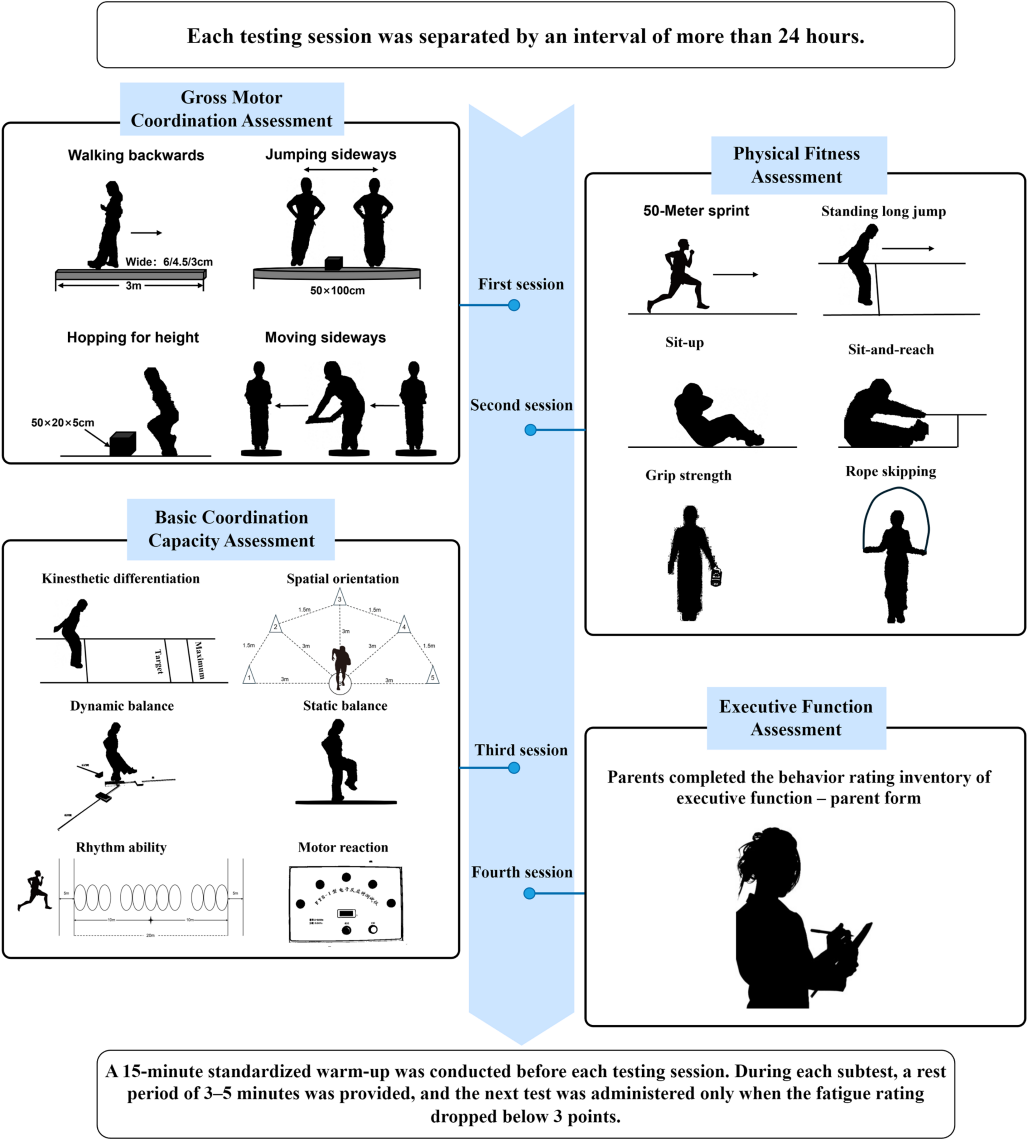
Test procedure.

All assessments were conducted between July and August 2023 in the gymnasiums and outdoor playgrounds of the participating schools. All tests were administered by trained sport-science examiners using standardized protocols, with each item consistently delivered by the same examiner to enhance reliability. To reduce bias from task unfamiliarity, participants received standardized briefings and demonstrations, and completed 2–3 familiarization trials per item to learn the required movement patterns and procedures, thereby improving measurement reliability. On each testing day, participants completed a 15-min standardized warm-up consisting of light jogging, core activation, and dynamic stretching. Subsequently, the following four test categories were administered in a fixed sequence: (1) Gross motor coordination, (2) Physical fitness, (3) Basic coordination capacities, and (4) Executive function. The executive function was assessed using the Behavior Rating Inventory of Executive Function (BRIEF, Parent Form), completed by participants’ primary caregivers. To minimize fatigue-related confounds, each test category was scheduled at least 24 h apart. Within each session, participants rested 3–5 min between subtests, during which perceived fatigue was rated using a subjective fatigue scale. Formal testing resumed only when the fatigue score was below 3. A schematic overview of the Test Procedure is shown in Fig. 1.

### Gross motor coordination assessment

GMC was assessed using the Körperkoordinationstest für Kinder (KTK), a standardized and reliable test battery for children (Kiphard & Schilling, 1974). The KTK consists of four subtests: (1) Walking Backwards: Participants walked backward three times on each of three balance beams with decreasing widths (6, 4.5, and 3 cm; each 3 m long). The number of successful steps (maximum eight steps per trial) taken before stepping off the beam was recorded. (2) Jumping Sideways: Within a 50 cm × 100 cm area, participants performed as many consecutive two-footed lateral jumps over a small obstacle as possible in 15 s. The total number of jumps was recorded. (3) Hopping for Height: Using incrementally stacked foam blocks (each 50 cm × 20 cm × 5 cm), participants attempted single-leg hops. The highest successfully cleared height was recorded. (4) Moving Sideways: Starting on one platform while holding another, participants moved laterally by transferring and stepping onto the platforms as many times as possible within 20 s, with the number of successful transfers recorded. Raw scores from the four subtests were converted to the Motor Quotient (MQ) using age and sex-specific Flemish norms provided in the KTK manual. The MQ served as the primary outcome measure of GMC in this study (Vandorpe et al., 2011).

### Physical fitness assessment

Physical fitness was assessed using items from the Youth Fitness International Test (YFIT) (Ortega et al., 2024), supplemented with additional evidence-based tests. The specific protocols are as follows: (1) 50-m sprint was used to evaluate sprint speed and anaerobic power. Timing gates were placed at the start and finish lines on a rubber track, with completion time recorded to the nearest 0.1 s (Hands, 2008). (2) Standing long jump assessed lower-limb explosive strength. Participants performed three maximal forward jumps from a standing position; the longest distance was recorded to the nearest 0.1 cm (Haga, 2009). (3) Sit-up measured muscular endurance. Participants lay on a mat with knees bent at 90°, feet secured, and hands placed on the chest or beside the ears. They performed as many correct repetitions as possible within 1 min; only sit-ups where the scapulae fully lifted off the mat were counted (Vaara et al., 2015). (4) The sit-and-reach test assessed flexibility using a standardized device (model PL-009-14A, Peilin, China). Participants sat with legs extended and pushed a sliding marker forward with their fingertips; the farthest distance reached was recorded to the nearest 0.1 cm. Three trials were performed, and the best result was used for analysis (Vaara et al., 2015). (5) Grip strength was measured using a digital hand dynamometer (Camry EH101, Xiangshan, China), adjusted according to hand size and sex. Grip strength is commonly used as a proxy for overall maximal muscular strength, particularly of the upper limbs. The best of two trials for each hand was recorded to the nearest 0.1 kg (Norman et al., 2011). (6) Rope skipping was used to assess coordination and aerobic capacity. Participants performed as many correct bilateral jumps as possible within 1 min; only successful jumps were counted toward the final score (Zhang et al., 2023).

### Basic coordination capacity assessment

Basic motor coordination refers to an individual’s ability to regulate timing, spatial orientation, and force during both simple and complex movements. Six subtests were used to assess this capacity (Sui et al., 2024). These subtests have been shown to possess high reliability and validity, and are particularly well-suited for evaluating BCC in children aged 9–10 years.

(1) Kinesthetic differentiation was evaluated using the target standing long jump test, in which the target was set at 70% of each participant’s maximal jump distance. Participants performed three jumps aiming to land as close as possible to the target, and the mean absolute deviation from the target was recorded (Đolo, Grgantov & Milić, 2019). (2) Spatial orientation was assessed *via* the numbered medicine ball running test. After three guided practice trials along a predefined route (see Fig. 1) and a 3 min rest, participants responded to verbal cues by sprinting from point B through a photoelectric timing gate toward designated marker cones, then returning to point B. The process was repeated three times, with the final trial requiring a second pass through the gate. Total time was recorded to the nearest 0.01 s (Peker & Vural, 2018). (3) Dynamic balance was assessed using the Y Balance Test. Leg length was measured supine from the anterior superior iliac spine (ASIS) to the most distal point of the medial malleolus, accurate to 0.5 cm. To eliminate the stabilizing effect of footwear, all tests were performed barefoot. During the Y Balance Test, participants stood on the central platform with both hands placed firmly on their hips and balanced on one leg, while reaching with the other leg in three directions—anterior, posteromedial, and posterolateral—to push the reach indicator as far as possible. After each reach, participants returned to the starting position while maintaining balance. Each direction was tested three times, and the maximum reach distance was recorded to the nearest 0.5 cm. The composite score was calculated by summing the maximum distances in all three directions, dividing by leg length, and averaging the scores of both legs for subsequent analysis (Fusco et al., 2020). (4) Static balance was tested under both eyes-open and eyes-closed conditions. Participants stood with hands on hips and lifted one leg 10–20 cm off the ground. Each trial lasted up to 60 s and was terminated if the hands or lifted leg touched the ground, the trunk swayed noticeably, or the support foot shifted. Each leg was tested three times per condition, and the average duration across both legs was used for analysis (Hands, 2008). (5) Rhythm ability was measured using the rhythmic sprint test. After completing two 30 m maximal sprints, participants ran through 11 rhythm rings arranged in a fixed pattern (see Fig. 1). Sprint times were recorded using timing gates. Rhythm score was calculated by subtracting the rhythmic sprint time from the best 30 m sprint time (Peker & Vural, 2018). (6) Motor reaction was assessed using an electronic reaction timer (FYS-II, Zhejiang Psychological Instrument Co., Hangzhou, China). Participants pressed a response button as quickly as possible following a photonic signal. Three trials were conducted, and the fastest response time was recorded to the nearest 0.01 s (Bańkosz, Nawara & Ociepa, 2013; Wang et al., 2006).

### Executive function assessment

Executive function (EF) was assessed using the Behavior Rating Inventory of Executive Function (BRIEF), Parent Form (Gioia et al., 2000). This questionnaire, completed by participants’ primary caregivers, consists of 86 items and yields three main scores: the Behavioral Regulation Index (BRI), the Metacognition Index (MI), and the Global Executive Composite (GEC). These indices are derived from eight subscales: Inhibit, Shift, Emotional Control, Initiate, Working Memory, Plan, Organize, and Monitor, which together evaluate core components of executive functioning in children aged 6 to 18 years. These scales index core components of EF in children aged 6–18 years. The Chinese BRIEF has demonstrated solid psychometrics, with test–retest reliability r = 0.68–0.89 and internal consistency α = 0.74–0.96, supporting acceptable temporal stability and internal reliability. Accordingly, the BRIEF is appropriate for assessing EF in school-aged children in China (Qian & Wang, 2007).

### Machine learning

Given the potential nonlinear relationships among variables, four machine learning algorithms known for their strong performance in nonlinear regression tasks were selected for model development and comparison: (1) Extreme Gradient Boosting (XGBoost): Regularized gradient-boosted trees, strong on tabular data; (2) Light Gradient Boosting Machine Regression (LGBM) Histogram, leaf-wise boosting—fast and scalable; (3) Random Forest Regression (RFR): An ensemble of bagged trees capturing nonlinearity and interactions; (4) Support Vector Regression (SVR): Margin-based regression; kernels enable nonlinear modeling. These algorithms have demonstrated high predictive accuracy and computational efficiency in handling complex patterns in high-dimensional feature spaces (Gu et al., 2015; Kensert et al., 2018; Mandorino et al., 2021; Paleczek et al., 2024).

### Feature selection and data preprocessing

The dataset was randomly split into a training set and a test set at a ratio of 4:1. To address multicollinearity, pairwise Spearman correlation coefficients were calculated among all variables in the training set, and variables with high correlation (R > 0.85) were excluded. The remaining variables were retained for further modeling. Since gender was included as a categorical feature, one-hot encoding was applied (Okada, Ohzeki & Taguchi, 2019). All features were standardized prior to model training. Additionally, Recursive Feature Elimination (RFE) was used in the training set to eliminate redundant variables and retain the 15 most informative features for subsequent training, aiming to reduce dimensionality and improve model performance (Zhao et al., 2021). To ensure the generalizability of the final results and considering that a very small test set can lead to unstable performance estimates, while an overly large test set reduces training data and weakens model development, we reserved 20% of the data for a stable final evaluation and used the remaining 80% exclusively for model building. Therefore, the 4:1 train-test split ratio was adopted to ensure that the test set was untouched and reserved for final evaluation, while the training set was used for feature selection and hyperparameter optimization.

### Model development, validation, and interpretation

**Figure 2:**
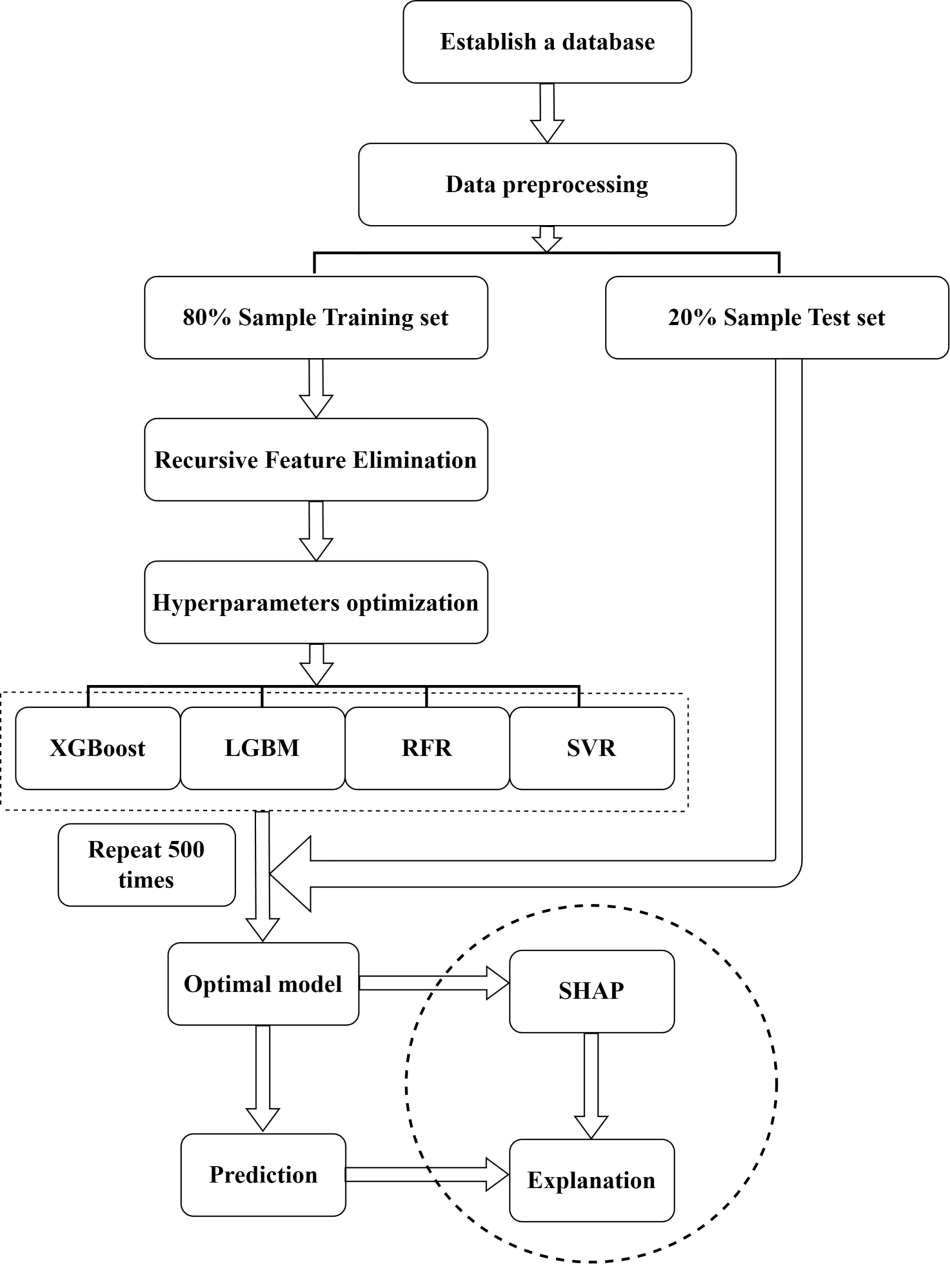
Flowchart of model training, evaluation, and SHAP value analysis.

Randomized search with 5-fold cross-validation was used to optimize the hyperparameters of the XGBoost, LGBM, RFR, and SVR models (Mandorino, Clubb & Lacome, 2024). Model performance on the test set was evaluated and compared using four metrics: coefficient of determination (*R^2^*), mean absolute error (MAE), and root mean squared error (RMSE). Given the common criticism that machine learning models function as “black boxes,” this study incorporated SHapley Additive exPlanations (SHAP), a game-theoretic approach proposed by Lundberg, Erion & Lee (2018), to enhance model interpretability. SHAP values were used to explain the predictions of the best-performing model and to identify the most influential features contributing to GMC (Lundberg, Erion & Lee, 2018). An overview of the end-to-end analytical pipeline—covering data preprocessing, feature selection and hyperparameter optimization, model training/evaluation, and SHAP-based interpretation—is shown in Fig. 2.

### Multiple linear regression analysis

In addition to the machine learning models, a multiple linear regression analysis using ordinary least squares (OLS) was conducted. After removing highly correlated variables, the remaining features were one-hot encoded and split into training and test sets. The regression model’s performance was evaluated using the same metrics: *R^2^*, MAE, and RMSE, to assess model fit and prediction error. All analyses were conducted using PyCharm Community Edition 2024.2.2, and the statistical significance level was set at *p* < 0.05.

## Results

### Feature selection

**Figure 3:**
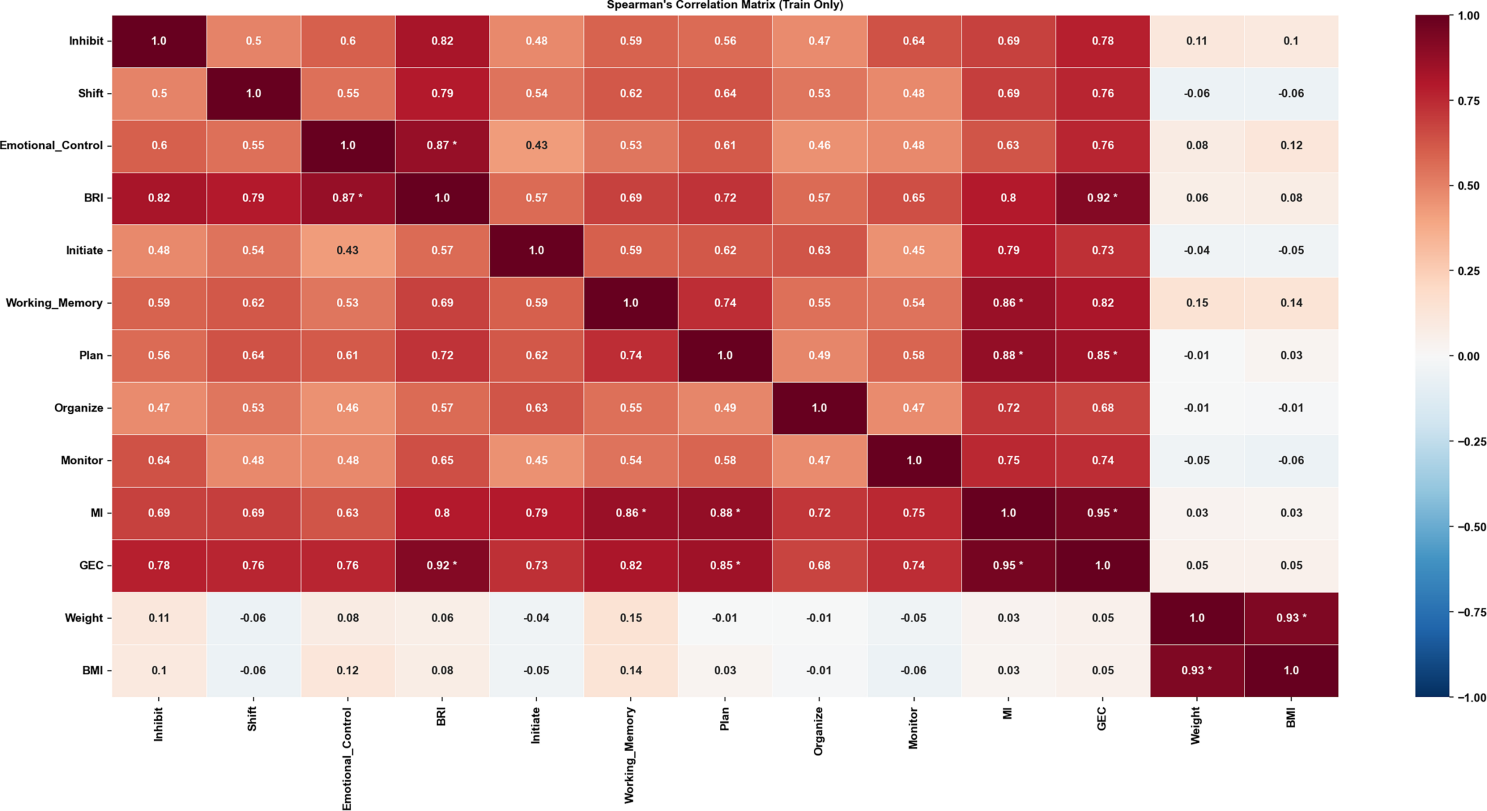
Spearman correlation (Train Only).

This study included 167 children aged 9–10 years (85 boys, 82 girls; height: 1.41 ± 0.74 m; weight: 36.89 ± 9.02 kg; Table 1). Overall GMC levels were comparable between boys and girls (86.63 ± 9.29 *vs*. 87.68 ± 9.39; Table 2). In terms of physical fitness, boys showed slightly higher performance in standing long jump and grip strength (131.79 ± 22.38 cm; 14.80 ± 2.56 kg), whereas girls performed better on the sit-and-reach (13.43 ± 4.96 cm) and completed slightly more rope-skipping repetitions (123.29 ± 30.76). For basic coordination capacities, girls exhibited superior static balance, with longer durations in the eyes-open and eyes-closed single-leg stance tests (56.91 ± 17.05 s; 16.61 ± 16.13 s). Building on the descriptive profiles, we prepared variables for modeling by computing pairwise Spearman correlations in the training set (full matrix in Table S2; key results in Fig. 3). The BRIEF indices BRI, MI, and GEC were highly correlated with the eight subscales (R > 0.85) and were therefore excluded. Similarly, body weight was removed due to its high correlation with BMI (R = 0.93). All remaining variables were retained for subsequent analyses.

**Table 2:** Descriptive statistics of included variables.

	Variable	Male (*n* = 85)	Female (*n* = 82)	Total (*n* = 167)
Gross motor coordination	Walking backwards	75.39 ± 14.37	81.72 ± 16.74	78.64 ±15.90
	Jumping sideways	108.48 ±11.57	105.38 ± 14.04	106.96 ±12.90
	Hopping for height	82.14 ± 11.14	81.03 ±11.63	81.57 ±11.37
	Moving sideways	81.62 ± 11.50	82.24 ± 8.98	81.93 ±10.31
	Motor quotient	86.63 ± 9.29	87.68 ± 9.39	87.14 ± 9.32
Physical fitness assessment	50-m sprint	10.32 ± 0.82	10.29 ± 0.62	10.30 ± 0.73
	Standing long jump (cm)	131.79 ± 22.38	126.85 ± 17.90	129.37 ± 20.39
	Sit-up (repetitions)	28.27 ± 7.25	28.85 ± 6.79	28.56 ± 7.01
	Sit and reach (cm)	7.90 ± 5.25	13.43 ± 4.96	10.61 ± 5.80
	Grip strength (kg)	14.80 ± 2.56	13.68 ± 2.49	14.25 ± 2.58
	Rope skipping (repetitions)	118.96 ± 36.19	123.29 ± 30.76	121.09 ± 33.61
Basic coordination capacity assessment	Kinesthetic differentiation (cm)	6.55 ± 2.65	6.83 ± 3.46	6.69 ± 3.07
	Spatial orientation (s)	11.73 ± 1.21	11.97 ± 1.30	11.85 ± 1.26
	Dynamic balance	0.88 ± 0.10	0.90 ±0.09	0.90 ± 0.09
	Eyes-open static balance (s)	37.85 ± 18.36	56.91 ± 17.05	42.24 ± 18.24
	Eyes-closed static balance (s)	10.14 ± 10.20	16.61 ± 16.13	13.32 ± 13.79
	Rhythm ability (s)	2.21 ± 0.56	2.59 ± 0.53	2.40 ± 0.57
	Motor reaction (s)	0.51 ± 0.19	0.50 ± 0.05	0.50 ± 0.14
Executive function assessment	Inhibit	12.59 ± 2.63	11.89 ± 2.14	12.25 ± 2.42
	Shift	10.80 ± 2.21	10.70 ± 1.87	10.75 ± 2.04
	Emotional control	12.98 ± 2.96	13.10 ± 2.89	13.04 ± 2.91
	Initiate	10.80 ± 2.13	10.52 ± 1.87	10.66 ± 2.00
	Working memory	13.27 ± 2.74	13.26 ± 2.60	13.26 ± 2.67
	Plan	16.07 ± 3.12	15.73 ± 2.91	15.90 ± 3.01
	Organize	7.84 ± 1.80	7.59 ± 1.64	7.71 ± 1.72
	Monitor	11.60 ± 2.40	11.20 ± 2.42	11.40 ± 2.41
	Behavioral regulation index	36.36 ± 6.49	35.68 ± 5.71	36.03 ± 6.11
	Metacognition index	59.58 ± 10.01	58.29 ± 9.47	58.95 ± 9.74
	Global executive composite	113.89 ± 18.78	111.74 ± 17.75	112.83 ± 18.26

**Note:**

Values are presented as mean ± standard deviation.

### Multiple linear regression results

The results of the multiple linear regression analysis are presented in Table 3. The model demonstrated a moderate level of fit (*R^2^* = 0.558; adjusted R^2^ = 0.465), with a SEE of 11.299, a MAE of 4.782, and a RMSE of 6.128.

**Table 3:** Model fit of multiple linear regression predicting gross motor coordination from physical fitness, basic coordination, and executive function.

*R* ^*2*^	Adjusted *R*^*2*^	SEE	MAE	RMSE	DW
0.558	0.465	11.299	4.782	6.128	1.651

**Note:**

*R*^*2*^, coefficient of determination; SEE, Standard Error of the Estimate; MAE, Mean Absolute Error; RMSE, Root Mean Squared Error; DW, Durbin-Watson test.

No multicollinearity issues were detected, as all variance inflation factor (VIF) values for the included predictors were below 5. Further examination of the regression coefficients (Table 4) revealed that sit-and-reach (β = 1.6697, *p* = 0.044), spatial orientation ability (β = –1.7148, *p* = 0.020), and eyes-closed static balance (β = 1.3938, *p* = 0.050) had statistically significant effects on gross motor coordination.

**Table 4:** Regression coefficients of multiple linear regression model predicting gross motor coordination from physical fitness, basic coordination, and executive function.

	β	SEE	T	*P*	95% CI	VIF
BMI	−1.442	0.832	−1.734	0.086	[−3.090 to 0.206]	1.974
Gender	−0.181	0.857	−0.211	0.833	[−1.880 to 1.517]	2.153
50-meter sprint	−0.771	0.924	−0.835	0.406	[−2.603 to 1.060]	2.421
Standing long jump jump	0.982	0.984	0.999	0.320	[−0.967 to 2.932]	2.731
Sit-up	0.734	0.723	1.016	0.312	[−0.698 to 2.166]	1.624
Sit and reach	1.670	0.821	2.035	0.044*	[0.043–3.296]	1.987
Grip strength	−0.625	0.85	−0.735	0.464	[−2.310 to 1.060]	2.089
Rope skipping	0.768	0.755	1.018	0.311	[−0.728 to 2.264]	1.645
Kinesthetic differentiation	−0.942	0.711	−1.326	0.188	[−2.351 to 0.466]	1.340
Spatial orientation	−1.715	0.726	−2.362	0.020*	[−3.154 to −0.276]	1.571
Dynamic balance	−0.841	0.718	−1.172	0.244	[−2.264 to 0.581]	1.465
Eyes-open static balance	1.504	0.771	1.95	0.054	[−0.025 to 3.033]	1.648
Eyes-closed static balance	1.394	0.703	1.984	0.050*	[0.001–2.786]	1.471
Rhythm ability	−0.256	0.957	−0.267	0.790	[−2.153 to 1.641]	2.837
Motor reaction	−1.279	0.817	−1.565	0.120	[−2.898 to 0.341]	1.863
Inhibit	−0.256	0.957	−0.267	0.790	[−2.153 to 1.641]	2.837
Shift	−0.181	0.857	−0.211	0.833	[−1.880 to 1.517]	2.154
Emotional control	−1.182	0.941	−1.256	0.212	[−3.048 to 0.684]	2.599
Initiate	0.372	0.869	0.428	0.669	[−1.351 to 2.096]	2.143
Working memory	−0.442	1.176	−0.375	0.708	[−2.773 to 1.890]	3.678
Plan	−0.151	1.173	−0.129	0.898	[−2.476 to 2.173]	3.857
Organize	0.262	0.907	0.288	0.774	[−1.536 to 0.059]	2.211
Monitor	0.054	0.879	0.062	0.951	[−1.688 to 1.797]	2.091

**Note:**

* Represents significance; β, Regression coefficient; SEE, Standard Error of the Estimate; 95% CI, 95% Confidence Interval; VIF, Variance inflation factor.

### Machine learning analysis results

In this study, model performance was assessed comprehensively by considering higher R^2^ together with lower RMSE, and MAE to ensure both predictive accuracy and reliability of feature interpretation. Among the four models tested, the RFR model achieved the best overall performance (*R*^2^ = 0.533; RMSE = 6.075; MAE = 4.850). Both XGBoost (*R*^2^ = 0.430; RMSE = 6.710; MAE = 5.233) and SVR (*R*^2^ = 0.489; RMSE = 6.360; MAE = 4.682) demonstrated comparable predictive ability and reasonable generalization capacity. In contrast, LGBM showed relatively weaker performance (*R*^2^ = 0.432; RMSE = 6.695; MAE = 5.525) compared to the other three models (Table 5). To further examine the stability of the RFR model, 5-fold cross-validation was performed, yielding results of *R*^2^ = 0.353 ± 0.155; RMSE = 7.148 ± 0.928; MAE = 5.586 ± 0.795. Although some fluctuation was observed, the model demonstrated generally good generalization. Given its superior predictive performance, the RFR model was selected for subsequent interpretation of key predictors.

**Table 5:** Model fit indices of four machine learning models.

	*R^2^*	95% CI	RMSE	95% CI	MAE	95% CI
XGBoost	0.430	[0.0736–0.6273]	6.710	[4.9626 to 8.4375]	5.233	[3.9053 to 6.7491]
LGBM	0.432	[0.1049–0.6018]	6.695	[5.2286 to 8.1055]	5.525	[4.3210 to 6.8340]
RFR	0.533	[0.2331–0.6498]	6.075	[4.7677 to 7.5947]	4.850	[3.7919 to 6.2757]
SVR	0.489	[0.1942–0.6786]	6.360	[4.4156 to 8.1499]	4.682	[3.3225 to 6.2368]

**Note:**

XGBoost, Extreme Gradient Boosting; LGBM, Light Gradient Boosting Machine; RFR, Random Forest Regression; SVR, Support Vector Regression; *R*^*2*^, coefficient of determination; 95% CI, 95% Confidence Interval; MAE, Mean Absolute Error; RMSE, Root Mean Squared Error.

### Feature importance and variable interpretation

**Figure 4:**
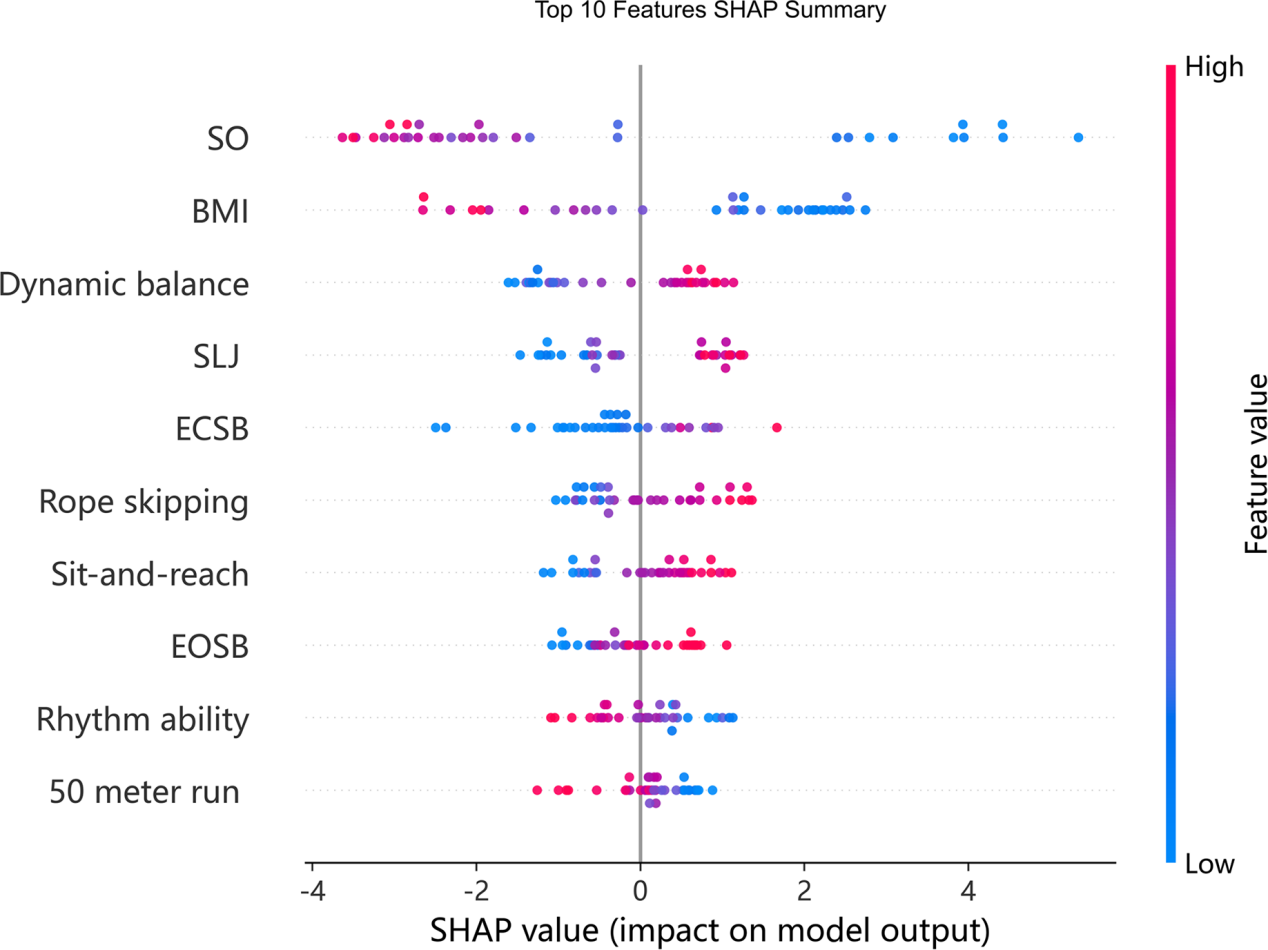
Summary plot of the random forest model.

According to the feature contributions derived from the RFR model (Fig. 4), spatial orientation, BMI, rhythm ability, and 50-m sprint performance made negative contributions to gross motor coordination. In contrast, dynamic balance, standing long jump, eyes-closed static balance, rope skipping, sit-and-reach, and eyes-open static balance made positive contributions. In the figure, negative SHAP values represent negative impacts, while positive SHAP values represent positive impacts on gross motor coordination.

**Figure 5:**
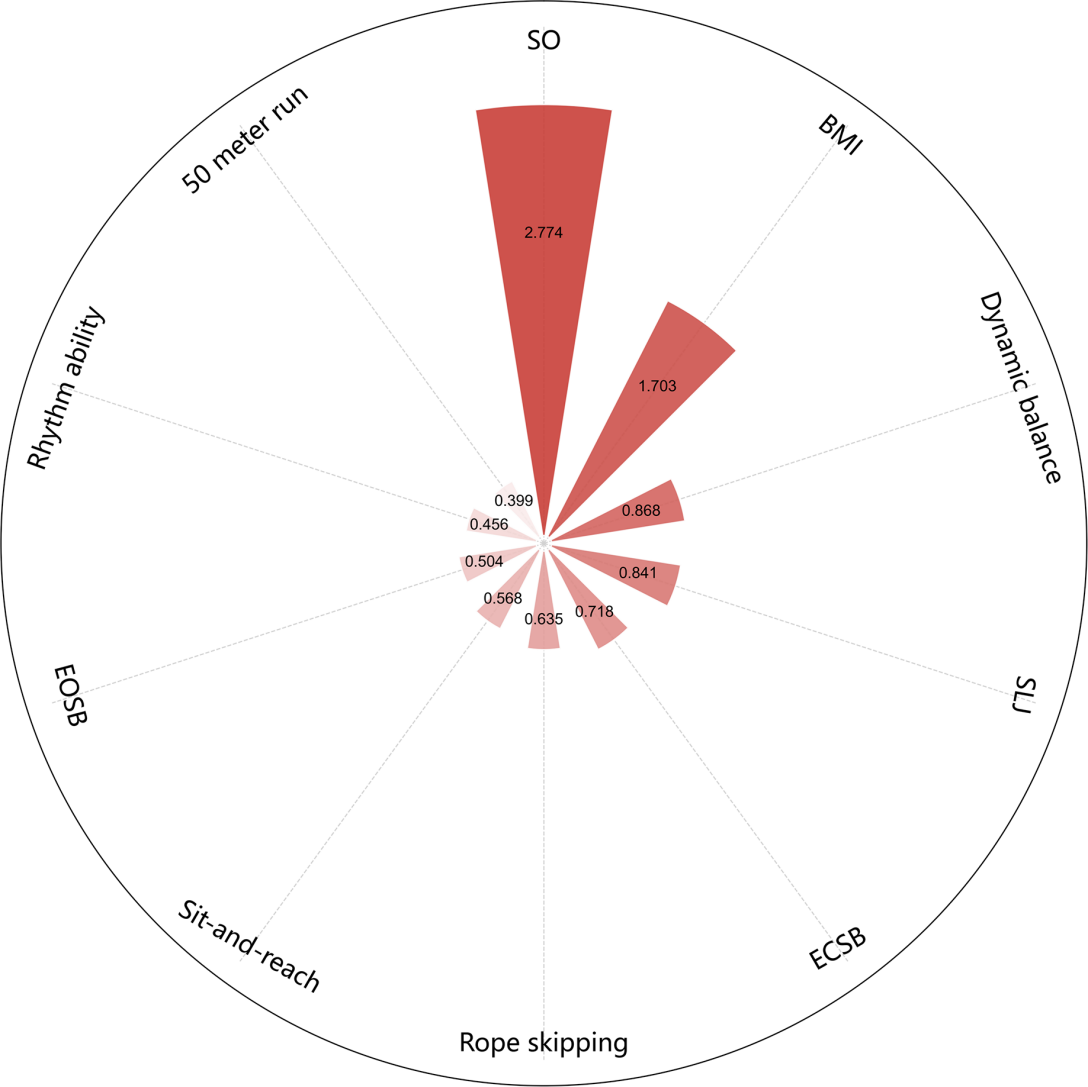
Feature importance plot of the random forest model.

Figure 5 displays the ranked importance of all features. The top ten contributors in the RFR model were, in descending order: spatial orientation ability, BMI, dynamic balance, standing long jump, eyes-closed static balance, rope skipping, sit-and-reach, eyes-open static balance, rhythm ability, and 50-m sprint. Notably, spatial orientation ability and BMI had substantially higher importance scores compared to the remaining variables.

**Figure 6:**
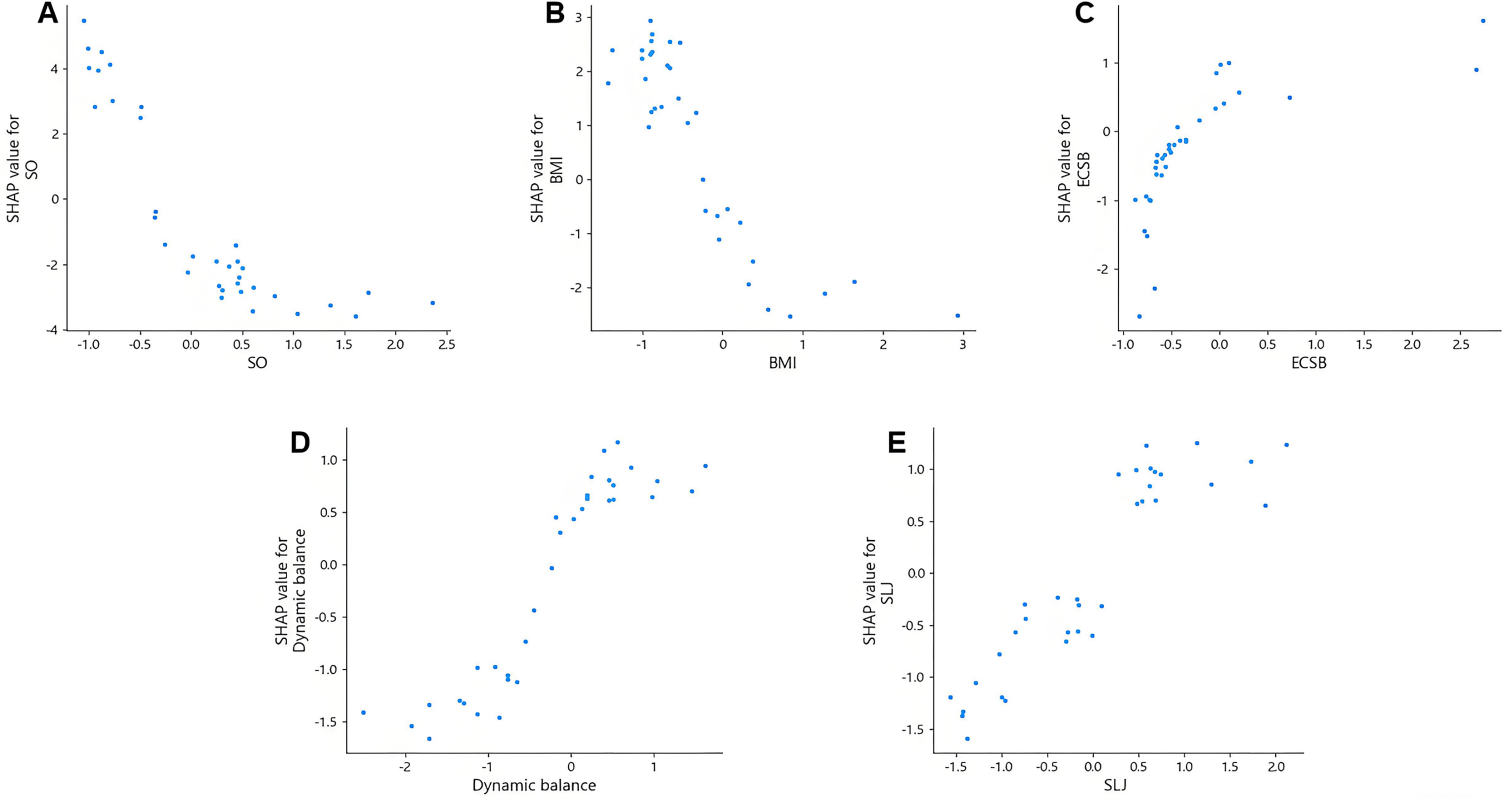
SHAP dependence plots.

Figure 6 presents SHAP dependence plots for the top five features—spatial orientation, BMI, dynamic balance, standing long jump, and eyes-closed static balance—to explore potential non-linear relationships with gross motor coordination. Overall, spatial orientation, eyes-closed static balance, and BMI exhibited clearly non-linear associations, whereas dynamic balance and standing long jump demonstrated approximately linear patterns.

## Discussion

This study compared four machine learning models—XGBoost, LGBM, RFR, and SVR—with a traditional multiple linear regression model to investigate the effects of physical fitness, basic coordination capacities, and executive function on GMC in children, aiming to construct an optimal predictive framework. Among all models, the RFR achieved the best predictive performance. We then applied SHAP to the best model for interpretability and identified the top ten contributors to GMC. The findings indicate that physical fitness and basic coordination capacities were the most influential predictors; notably, spatial orientation, eyes-closed static balance, and BMI showed nonlinear relationships with GMC, whereas executive function had a comparatively smaller impact.

The multiple linear regression model achieved an adjusted R^2^ of 0.465, with spatial orientation ability, sit-and-reach, and static balance (eyes closed) showing significant effects on GMC (*p* < 0.05), consistent with previous findings (De Chaves et al., 2016; Sui et al., 2024). However, compared to the machine learning models, the linear model exhibited relatively larger prediction errors (SEE = 11.299, RMSE = 6.128, MAE = 4.782), suggesting limited generalizability and stability. This may be attributed to constraints such as variable selection, sample characteristics, or the linear model’s inability to capture complex, nonlinear interactions. In contrast, the RFR model outperformed others, yielding a higher *R*^2^ (0.533) and lower prediction errors (RMSE = 6.075, MAE = 4.850), demonstrating superior predictive performance. These findings suggest that machine learning models—particularly RFR—are better suited to handling high-dimensional and nonlinear data in predicting children’s motor performance. Furthermore, the SHAP-based analysis strengthened the model’s interpretability by providing accurate and reliable evaluations of feature contributions and relative importance.

Compared to the linear regression model, this approach identified additional influential variables. Specifically, spatial orientation, BMI, dynamic balance, standing long jump, and eyes-closed static balance emerged as the top five predictors, highlighting the essential roles of spatial awareness, body composition, lower-limb strength, and both static and dynamic balance in GMC development. As an important indicator of body composition, BMI has been widely recognized as a significant factor affecting GMC and motor competence in children (D’Hondt et al., 2014; Fernandes et al., 2022; Hudson, Ballou & Willoughby, 2021). Excess adiposity increases mechanical load and reduces movement economy, which can constrain coordination; conversely, a healthy weight supports neuromuscular efficiency and movement control, facilitating better coordination (Biino et al., 2023; Fernandes et al., 2022).

Among all predictors, spatial orientation ability showed the strongest impact on GMC and demonstrated a nonlinear association. Previous studies have emphasized the close connection between spatial orientation and rhythm ability, noting their mutually reinforcing roles in sustained physical activity (Sui et al., 2024; Qu, 2020). Rhythm training not only supports spatial perception, postural control, and dynamic balance but also improves multisensory integration, thereby enhancing motor coordination and execution efficiency (Jerzy et al., 2015; Peker & Vural, 2018). Notably, rhythm ability also ranked among the top ten contributing factors to gross motor coordination in this study. Although empirical research specifically addressing the impact of spatial orientation on GMC remains limited, prior evidence suggests significant correlations between spatial orientation, rhythm ability, and motor coordination (Hands, 2008; Jerzy et al., 2015). Therefore, it can be inferred that spatial orientation may be one of the key factors influencing the development of GMC. Systematic training targeting spatial orientation may help enhance children’s body-space awareness, information processing, and performance in complex motor tasks (Boccia et al., 2017). In addition, Dynamic balance, eyes-closed static balance, and standing long jump also demonstrated strong predictive power in the model, reinforcing their importance in maintaining posture and executing coordinated movements. The observed higher predictive value of eyes-closed static balance compared to eyes-open balance may stem from the increased reliance on proprioceptive and vestibular input in the absence of visual cues. These sensory systems are essential for maintaining balance during complex multi-joint and multi-degree-of-freedom movements, which often require precise sensory feedback and tightly coordinated isometric, concentric, and eccentric muscle actions (Dos Santos et al., 2018; Jain et al., 2022). Notably, these variables were not prominent in the linear regression model but showed strong explanatory power in the RFR model, reinforcing the advantage of ML in detecting nonlinear interactions and latent influential features.

Notably, executive function did not emerge as a significant predictor of GMC in this study. Effective engagement of executive function typically requires novel, cognitively demanding tasks that activate the cerebellar–prefrontal circuitry (Ren et al., 2025). In this study, executive function was assessed using the parent-reported BRIEF questionnaire, which, while practical and easy to administer, may not fully capture children’s cognitive regulation during actual movement or task-based scenarios. Subjective assessments can be influenced by parental interpretation bias and fragmented observation, potentially underestimating the true role of executive function. Future studies are encouraged to employ more objective assessments—such as behavioral tasks combined with neuroimaging techniques (*e.g*., fNIRS or EEG)—to gain deeper insight into the contribution of executive function to GMC in children.

In summary, the findings suggest that physical fitness and basic coordination capacities exert a stronger influence on GMC than executive function, and demonstrate greater predictive value. Therefore, early intervention strategies should focus on enhancing spatial orientation, increasing physical activity to reduce BMI, and improving lower-limb strength as well as both static and dynamic balance. Specific recommendations include using maze navigation and orienteering games to develop spatial awareness and directional judgment; using rhythmic activities such as rope skipping, rhythmic gymnastics, and patterned running to develop children’s sensitivity to movement rhythm and improve their motor coordination and fluidity; and combining dynamic/static stretching and plyometric training to improve muscle strength, flexibility, and postural stability (Ayotte & Corcoran, 2018; Hands, 2008), thereby facilitating overall GMC development.

While this study presents a relatively comprehensive model, several limitations should be noted. The cross-sectional design and omission of contextual/psychosocial variables limit causal inference and interpretability. A modest sample from three Shanghai public schools without school-/sex-stratified anan this study, model performance was assessed comprehensivelylyses may reduce generalizability. EF was measured only by parent-report (BRIEF), which could underestimate EF–GMC associations. Despite these limitations, this study was among the first to integrate physical fitness, basic coordination, and executive function using machine learning techniques to examine the underlying factors contributing to gross motor coordination in children. Future research should consider longitudinal or intervention-based designs to establish causal pathways, expand the variable framework to include ecological and psychological dimensions, and adopt multimodal assessment approaches to improve the validity and generalizability of findings.

## Conclusions

This study show that the RFR model outperformed traditional linear regression in predicting children’s GMC, suggesting the presence of complex nonlinear relationships that ML methods capture more effectively. SHAP analysis identified spatial orientation, BMI, dynamic balance, standing long jump, and eyes-closed static balance as the most influential predictors, with spatial orientation, eyes-closed static balance, and BMI showing clear nonlinear effects, whereas EF contributed little. Overall, RFR both improves prediction and identifies actionable targets—namely spatial awareness, balance, lower-limb strength, and healthy body composition.

## Supplemental Information

10.7717/peerj.20827/supp-1 Supplemental Information 1 Dataset.

10.7717/peerj.20827/supp-2 Supplemental Information 2 Full matrix.

10.7717/peerj.20827/supp-3 Supplemental Information 3 Light Gradient Boosting Machine Regression.

10.7717/peerj.20827/supp-4 Supplemental Information 4 Extreme Gradient Boosting.

10.7717/peerj.20827/supp-5 Supplemental Information 5 Support Vector Regression.

10.7717/peerj.20827/supp-6 Supplemental Information 6 Random Forest Regression.

## Additional Information and Declarations

### Data Availability

The following information was supplied regarding data availability:

The data and code are available in the Supplemental Files.

The code is available at Zenodo: Mao. (2025). code. Zenodo. https://doi.org/10.5281/zenodo.17060967.
